# Targeted Genetic Education in Dentistry in the Era of Genomics

**DOI:** 10.3390/genes15121499

**Published:** 2024-11-22

**Authors:** Farah Asa’ad, Anne Nørremølle, Qalbi Khan, Lena Larsson, Niels Tommerup, Nuno Vibe Hermann, Asli Silahtaroglu

**Affiliations:** 1 Department of Oral Biochemistry, Institute of Odontology, The Sahlgrenska Academy at University of Gothenburg, P.O. Box 450, SE 405 30 Göteborg, Sweden; farah.asaad@gu.se (F.A.); lena.larsson@odontologi.gu.se (L.L.); 2 Department of Cellular and Molecular Medicine, Faculty of Health and Medical Sciences, University of Copenhagen, Blegdamsvej 3, DK-2200 Copenhagen, Denmark; annenoe@sund.ku.dk (A.N.); ntommerup@sund.ku.dk (N.T.); 3 Institute of Oral Biology, Faculty of Dentistry, University of Oslo, Postboks 1052, Blindern, 0316 Oslo, Norway; qalbi.khan@odont.uio.no; 4 Department of Odontology, Faculty of Health and Medical Sciences, University of Copenhagen, Nørre Alle 20, DK-2200 Copenhagen, Denmark; nuno@sund.ku.dk

**Keywords:** dentistry, teeth, learning goals, teaching genetics, dental genetics, genomics, case study, next-generation sequencing

## Abstract

**Background:** The growing body of knowledge on the human genome and its variants points towards the significance of genetic factors in oral health and disease. Since the dental curricula have historically prioritized clinically oriented subjects, this focus has resulted in insufficient coverage of genetics. To leverage this knowledge in patient care, dental education must equip students with an understanding of the principles of genetics. **Method**: We have established “Genetic Educators Network in Dentistry” (GEN-Dent) to identify common concerns regarding genetics in dental education and work for a greater emphasis on genetics in future dental programs to make sure that professionals in dentistry are well-prepared to navigate the complexities of the evolving “human genome era”. **Results:** Here, GEN-Dent proposes specific learning goals for medical genetics in dentistry and provides supporting teaching material addressing each learning goal. The five life-like case studies exemplify different dental conditions and introduce important concepts of genetics, inspiring other educators. **Conclusions**: Opportunities in Scandinavian countries can be an advantage in increasing global awareness of the importance of genetics in dentistry. The integration of genetics into dental education not only aims to improve patient care but also seeks to inspire a new generation of basic scientists with clinical backgrounds in dentistry. We expect that using life-like patient cases will significantly motivate dental students when learning medical genetics.

## 1. Introduction

The worldwide efforts to sequence millions of genomes, find novel disease-causing genes, and develop tools for interpreting gene variants constantly increase the demand for the understanding of genetics among all healthcare disciplines. The understanding of genetic factors and their interactions with environmental factors is crucial for the future clinicians that aim to translate from the lab bench to bedside and to use this knowledge to ensure the well-being of their patients [1]. There is an urgent need to integrate genetics in the curriculum of all healthcare educations, including dental education, which engages the students and prepares them for a future, where genetic screening programs and information will be widely available due to emerging innovative solutions based on next generation sequencing.

Dentistry as a clinical discipline represents its unique challenges and demands, requiring a tailored approach to genetics education. A basic genetic education is necessary, highlighting the importance of the hundreds of currently identified genes involved in the development of the teeth, mouth, and face. The rapid advancements in biomedical sciences, such as genomics, microbiome research, systems biology, complexity sciences, and bioinformatics, highlight the need for dental education to align with these emerging fields to improve precision in public oral health and healthcare delivery [2]. Genetic predisposition and/or monogenic variants with direct causality are linked to various oral conditions, including dental developmental anomalies such as tooth agenesis and different types of mineralization disturbances [3]. More recently, periodontal diseases were also associated with genetic factors influencing the oral microbiome [4]. It is thus important for the students to understand the main concepts of genetics as well as to gain the ability to apply this knowledge to dentistry, through relevant cases on both common and rare dental diseases and related syndromes.

Understanding the genetic mechanisms and specific factors facilitates the application of precision medicine and personalized dental treatments tailored to individual genetic profiles, thereby improving clinical outcomes [5,6,7]. Teaching dental students about heritability and population-based genetic risk factors in dental diseases as well as epigenetic effects of social risk factors on common dental conditions will eventually have a beneficial impact on oral health efforts at the family, population, and even global level. For example, a dentist needs to be able to recognize heritability of the dental phenotype of their patients, draw and read a pedigree, and think about the relevant genetic testing options available [8]. Recent efforts to improve dental curricula worldwide have focused on emerging fields such as preventive dentistry [9], regenerative dentistry [10], geriatric dentistry [11], and even digital dentistry [12]. Despite these advancements, genetics remains insufficiently covered in many university dental programs. In our experience, there is a great variation in genetic education of dental students both in Scandinavia and other parts of the world [8,13].

However, integrating genetics into dental education presents several challenges. The continuous influx of new bioscience knowledge demands a reassessment of teaching methods [14]. The packed nature of dental curricula, integrating basic sciences, laboratory work, and clinical training, creates challenges for expanding the amount of genetics to be included in dental education [15]. Furthermore, the traditional separation of basic sciences from clinical training often limits a cohesive educational approach [9]. Issues such as shortage of trained faculty have been reported in many disciplines, further complicating the integration [16,17]. Additionally, infrastructure limitations and educational gaps can hinder the widespread adoption of genomics and related technologies in clinical practice [18]. The transition of genetics from research to clinical settings also raises concerns about confidentiality, ethics, and the effective application of genetic information [19].

To close the gap between basic science and clinical practice, we suggest using teaching methods based on life-like case stories mimicking clinical scenarios. Close collaboration between the teachers of basic sciences and clinical sciences could also make things flow smoothly over different semesters to increase the knowledge of both teachers and learners. We encourage the use of dental-related genetics databases, which provide valuable tools for enhancing genetics education and engaging dental students more effectively [20,21,22].

### All These Ideas Are True and Fine, How Do We Move Forward from Here?

While genetics education has been applied across various disciplines, its broad integration and acceptance of importance into dentistry is currently lacking. To address this gap, we have established the Scandinavian “Genetic Educators Network in Dentistry” dedicated to advocating for the inclusion of comprehensive genetics teaching in dental programs. We aim to influence the educators and study boards responsible for improving the dental curricula in the universities to include comprehensive genetics in education.

As educators of genetics at different dental study programs, one common thing we observe is that despite the different circumstances and different study program structures, we all share the same wish to increase the motivation among dental students to learn about genetics. In our experience, and the experience of others, using designed life-like patient cases in the teaching, thereby tailoring the education according to the expectations of future dentists, has a strong motivating effect on the students [23,24,25,26]. As an initial step, we would like to inspire the teachers, course organizers, and study boards by sharing examples of dental-related genetics patient cases with specific learning goals that could be incorporated into any dental education.

## 2. Learning Goals and Suggestions for Cases in Dental Genetics

We have formulated the following learning goals for medical genetics for dentistry, which covers the relevant objectives for what a future dentist in our opinion needs to know about genetics (Table 1). These learning goals can form the basis for a specific medical genetics course, or they can be implemented within different relevant courses.

The overarching theme of these learning goals is to prepare students of dentistry for a future where the genetic information of their patients would be even more detailed than today. The aim is to increase awareness of the importance of genetics in oral health and diseases to enable future dentists to put this genetic information into use. This goal can only be achieved by providing the students with an in-depth understanding of the basic concepts of genetics and the interplay between genotype and dental phenotype. Another key point of the learning goals is that they are seen from the perspective of what a dentist may encounter in their patients. This emphasizes that teaching should start from life-like situations, which is why we suggest using case stories designed to mimic situations in which a dentist would need knowledge and understanding of medical genetics to provide the best treatment for the patient. Case-based learning has been used for teaching Medical Genetics at both the Dental and the Medical Schools at the University of Copenhagen for more than a decade. In our experience, the introduction of life-like case stories in teaching increases the student’s motivation for learning the concepts of genetics [23].

**Figure 1:**
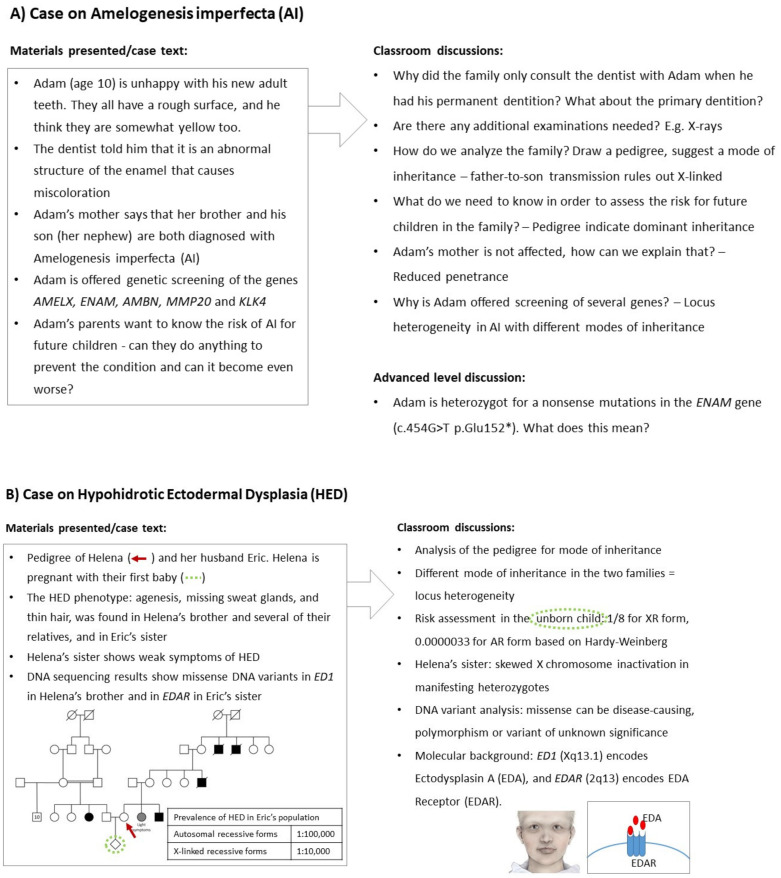

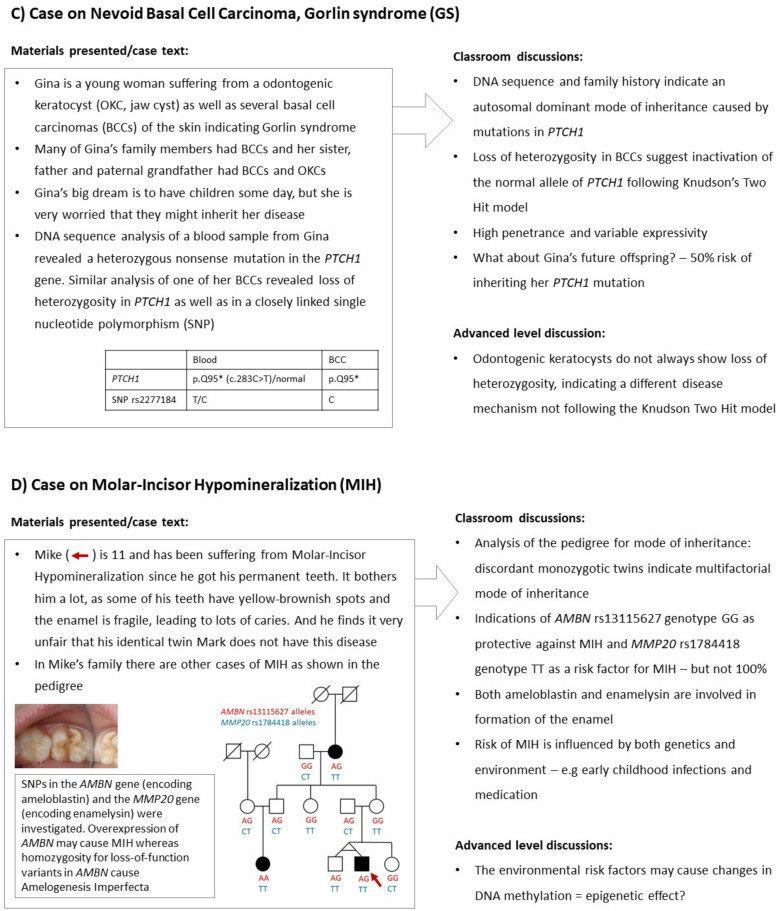

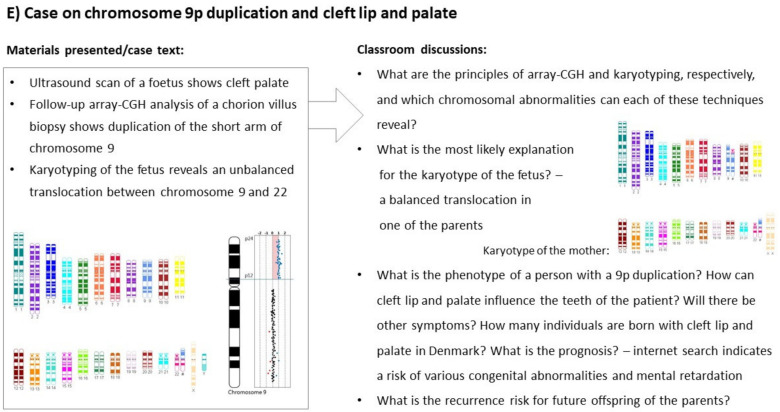
**Case suggestions for medical genetics for dentistry students.** Five examples of cases introducing key concepts of medical genetics through life-like patient stories, as they could be experienced in a dentist’s clinic or a hospital. (**A**) **Amelogenesis Imperfecta.** *Keywords: Autosomal Dominant, Reduced Penetrance, Locus Heterogeneity, Genes Regulating Enamel Formation, Nonsense Mutation.* The case introduces the students to drawing and analyzing a pedigree. Mode of inheritance is most likely autosomal dominant due to the phenotype appearing in father and son; however, this means that the mother of the proband presents reduced penetrance. A gene panel analysis is performed due to locus heterogeneity. The analysis reveals heterozygosity for a nonsense mutation in the *ENAM* gene as described in Seymen et al. 2014 [29]. (**B**) **Hypohidrotic Ectodermal Dysplasia.** *Keywords: Locus Heterogeneity, Autosomal Recessive, X-linked Recessive, Risk Assessment using Hardy–Weinberg, X-chromosome Inactivation, Genes Regulating Tooth Formation, Missense Mutation.* The pedigree of the proband indicates different modes of inheritance in her and her husband’s families. The risk assessment for the unborn child is performed for each HED form individually. The risk for the X-linked recessive form in the mother’s family is 1/8, as the maternal grandmother is a carrier, and only boys will be affected. The risk for the autosomal recessive form found in the father’s family is 0.0000033 as the carrier risk of the father is 2/3 and of the mother 0.00002 (based on Hardy–Weinberg calculation where q^2^ = 1/100,000 leads to 2 pq = 0.00002). The sister of the proband has a 0.5 risk of being a carrier. Non-random X-chromosome inactivation is most likely the reason why she presents with weak symptoms [30]. Illustration of patient by Malin Bernas-Theisen, TAKO-Center. (**C**) **Nevoid Basal Cell Carcinoma, Gorlin syndrome.** *Keywords: Hereditary Cancer of the Jaw and Skin, Autosomal Dominant, Knudson’s Two-Hit Model, SNP Linked to the Gene.* Gorlin syndrome (GS) is characterized by odontogenic keratocysts as well as early-onset of multiple basal cell carcinomas. GS may be caused by inherited loss-of-function mutations in the *PTCH1* gene as presented in the case. Family history indicates autosomal dominant mode of inheritance, which is confirmed by findings of heterozygosity for a nonsense mutation in *PTCH1* in the blood of the proband. Analysis of tumor tissue from the proband shows loss of heterozygosity for both the *PTCH1* mutation [31] as well as a closely linked single nucleotide polymorphism [32], indicating somatic deletion of the *PTCH1* region (including the SNP) as a second hit according to Knudson’s Two-Hit model. (**D**) **Molar–Incisor Hypomineralization.** *Keywords: Multifactorial Polygenic Inheritance, SNPs in Genes Regulating Enamel Formation, Discordant Monozygotic Twins.* The case story presents a family in which several cases of Molar–Incisor Hypomineralization (MIH) appear. Discordance for the phenotype in a pair of monozygotic twins indicates a multifactorial mode of inheritance. Analysis of two specific variants in the genes encoding ameloblastin and enamelysin suggests an association of the phenotype with the genotype indicating that these two genes are involved in the disease [33]. This may be linked to the role of the proteins encoded in the development of the enamel. Interestingly, loss-of-function DNA variants in the gene encoding ameloblastin *(AMBN)* may lead to Amelogenesis Imperfecta while increased expression of ameloblastin has been linked to Molar–Incisor Hypomineralization, allowing for discussion of the opposing effects of different DNA variants in the *AMBN* gene [34]. Environmental risk factors like early childhood infectious diseases might act through effecting the epigenetic regulation of gene expression [35]. (**E**) **Chromosome 9p duplication and cleft lip and palate.** *Keywords: Unbalanced Reciprocal Translocation, Cleft Lip and Palate.* A routine ultrasound scan of a fetus reveals a cleft lip and palate, which could be an indication of a chromosomal abnormality. Array-CGH and karyotyping of the fetus reveals an unbalanced 9;22 translocation leading to partial trisomy of the short arm of chromosome 9 (dup(9)(p12pter)). Discussion about the origin of this abnormality should lead to the understanding of a potential balanced translocation in one of the parents, indicating an increased recurrence risk in future offspring. A literature search will reveal a potential complex phenotype in the child [36], including a profound impact on teeth development. This specific case was developed as an interactive laboratory simulation in a collaboration between the University of Copenhagen and Labster ApS https://www.labster.com/. Karyograms were drawn using the CyDAS Package http://www.cydas.org/OnlineAnalysis/ accessed on 23 October 2024 [37].

Here, we present five different cases in which the students are introduced to different aspects of the learning goals (Figure 1). The cases are meant to be inspirational for teachers of medical genetics in dentistry. Through working with the cases, the students will be introduced to important concepts of medical genetics in dentistry including, e.g., monogenic disorders, modes of inheritance, allele and locus heterogeneity, X-chromosome inactivation, risk assessment including the Hardy–Weinberg principle, inherited cancer, multifactorial diseases, and different types of chromosomal variations. The five cases can be used in combination to provide coverage for all of the mentioned concepts, or individual cases can be used to enlighten specific concepts. At the University of Copenhagen, five different cases are used as the main material for classroom teaching, combined with lectures and supplemented with textbooks in medical genetics, to form the course in Medical Genetics for dentistry students. Later, in the clinical courses, students are again introduced to examples of genetic cases.

The cases can be presented to the students in a case-based learning or problem-based learning format [28]. In Figure 1, text and figures to the left are introduced to the students, either as a whole or in sequence, optionally accompanied by pictures of patients from the internet or publications. Student hand-outs are included as Supplementary Figures S1–S5. The information given is interpreted, analyzed, and discussed by the students in groups and/or classroom sessions. A teacher/tutor facilitates the process, using the list of questions/concepts to the right as input for the discussion when needed, and as a checklist to ensure that all the important aspects are covered. Ethical discussions can be included, if relevant, according to the study plan. Likewise, discussions on the methodology used for genetic testing may be incorporated. However, they should reflect the actual options available in the relevant country. The time and format used for the cases can vary—for example, a short in-class group discussion or a whole day session with the teacher giving information sequentially while letting the students discuss and present explanations and answers in between. The case text could be translated and modified to fit the circumstances in which they are used, just as the cases can be modified to form the basis for exam questions. For convenience, the figures used in the cases are presented as Supplementary Figures S6–S11.

The five cases presented here are designed to mimic real-life patient stories to enhance the motivation of the students while at the same time urging them to have a deeper understanding of the underlying concepts. In particular, cases A and B both describe rare monogenic disorders of the teeth displaying locus heterogeneity, but while case A focuses on concepts of dominant inheritance with reduced penetrance, case B presents risk calculations using population frequencies. Case C introduces the inheritance of cancer, exemplified by inherited increased risk of basal cell carcinoma associated with odontogenic cysts in Gorlin syndrome. The case introduces students to Knudson’s Two-Hit model for inherited cancers while emphasizing the important role of the dentists in diagnosis of the tumors of the mouth. Case D describes an example of a multifactorial mode of inheritance, in which accumulation of cases in a family indicates a genetic component whereas discordance in monozygotic twins suggests an environmental factor. Case E, on the other hand, describes a case where a rare chromosomal abnormality causes congenital malformations, here cleft lip and palate. Of importance, this case shows a fetus with an unbalanced reciprocal translocation that is inherited from a healthy parent carrying this translocation in a balanced form.

In most cases, we have included data describing specific DNA variants found in the patients to prepare the students for a future where this kind of information would be readily available. This will allow the students to familiarize themselves with data from DNA sequencing techniques and give them the ability to interpret the consequences of different types of DNA variants.

It is fully intentional that some of the case stories represent aspects of medical genetics not only related to tooth development and dental health but also to symptoms involving other organs (e.g., skin cancer, risk of mental retardation) that traditionally are diagnosed by medical doctors. This is performed to allow enhanced interaction and mutual understanding between the students of medicine and dentistry, who are often taught together in shared Medical Genetics courses.

The five cases presented here can be used as examples or frameworks for designing additional or alternative cases, e.g., with specific relevance to the country in which the teaching takes place. For inspiration, we refer to reviews providing information on various genes and genetic mechanisms involved in tooth development and disease [38,39,40].

## 3. Discussion

Recent research has identified numerous genetic factors associated with tooth formation, tooth agenesis, and various dental conditions, with approximately four thousand relevant publications over the past two decades. However, the field of genetics is underrepresented in dental education [13]. Dental curricula worldwide tend to emphasize areas of traditional dental topics such as cariology, periodontology, endodontics, prosthodontics, and oral surgery with a limited focus on genetic and epigenetic factors. This gap is notable even in many industrial countries, where, despite their high GDP per capita and free higher education, dental programs have varying amounts of teaching hours on genetics integrated into their study plans.

In dentistry, genetics is primarily applied in the diagnosis and management of rare congenital dental diseases and syndromes. For example, in Denmark, the Danish Health Act [41] outlines provisions for the treatment of rare genetic conditions with referrals to specialized centers for complex cases. Lifelong free treatment of challenges related to or caused by the specific condition is granted based on the severity of the condition and its impact on oral health, whereas free-of-charge ordinary dental care for all inhabitants under the age of 22 is provided through the municipal dental care services. Similar solutions with government-supported specialized centers for rare conditions exist in Norway (which in addition also covers 75% of the costs for those between 21–26 years of age [42] and Sweden, with their health acts defining the criteria for specialized dental care coverage based on the severity and impact of conditions on dental health [43]. 

A well-known example of a rare genetically determined condition of the teeth is Amelogenesis Imperfecta (AI). AI is an inherited/congenital condition affecting the enamel of both the primary and permanent dentition. Both the clinical picture of the disease and the prevalence of AI vary a lot. The global prevalence has been suggested to be 0.1–0.5% [44,45]. The variation in clinical expression is considerable. There are more than 14 different clinical variants with enamel disturbances from a slight decrease in enamel maturation and mineralization to larger areas of hypoplasia and lack of enamel, as well as stria of combined healthy and disturbed enamel formation. The prevalence of the disease is most likely underestimated. The condition affects the permanent dentition more than the primary dentition. AI can be caused by mutations in many different genes, including *AMELX*, *ENAM*, *AMBN*, *ACP4*, *MMP20*, *KLK-4*, *FAM83H*, *WDR72*, *C4orf26*, *SLC24A4*, *LAMB3*, and *ITGB6*, as well as related miRNA genes [46].

Dentinogenesis imperfecta (DI) is, like AI, a rare genetically determined condition in the teeth. However, DI affects only the dentin. The clinical picture of DI shows translucent and discolored teeth (most often blue-grey or yellow-brown). DI causes missing adherence between enamel and dentin, which leads to, among other things, breakage, wear and tear, abscesses, and premature loss of teeth. Compared to AI, the prevalence of this condition is estimated to be extremely rare (about 1:100,000–200,000). DI affects the primary dentition more than the permanent dentition, and the teeth that are latest formed are less affected. It is inherited in an autosomal dominant pattern due to mutations in the dentine sialophosphoprotein gene (*DSPP*) [47].

In addition to rare diseases, more common conditions such as Molar–Incisor Hypomineralization (MIH), which typically presents with hypomineralization of a first permanent molar with or without the combination of hypomineralized permanent incisors affects globally about 12,9% of the population, although regional differences occur with reported prevalences as high as about 40%. As the development of incisors and molars takes place at the same time, the cause has usually been thought to be of systemic origin [48]. However, recent studies suggest that MIH could be influenced by both genetic predisposition and environmental factors, where genetic predisposition interacts with environmental exposures to manifest the condition [49].

Teeth agenesis (genetically introduced missing tooth bud and tooth) occurs relatively often in the population with a reported occurrence of 3–10% (not including missing of the third molars) [50,51,52]. For comparison, congenital heart disease, which is considered the most common major congenital anomaly (excluding oral anomalies), has an incidence of less than 1% worldwide [53].

The above-mentioned conditions are just examples, and we currently need to focus on the new opportunities given to us by technological development allowing the collection of vast amounts of genetic information about most dental and oral-related conditions and diseases. Public health initiatives like nation-wide health registries containing dental information [54], when combined with increasing amounts of genomic sequence data in national genome centers, will constitute important resources for moving forward with the research on genetics of dental development and disease.

The World Health Organization (WHO) has currently recognized oral disease as a high-priority area until 2030 and called for urgent action https://cdn.who.int/media/docs/default-source/ncds/mnd/oral-health/eb152-draft-global-oral-health-action-plan-2023-2030-en.pdf?sfvrsn=2f348123_19%26download=true accessed on 23 October 2024. Efforts to enhance awareness of the genetic basis of dental diseases align with this global priority. Integrating genetic education into the dental curriculum can improve the understanding and management of dental conditions, ultimately benefiting patient care and treatment outcomes.

## 4. Conclusions

There is a growing insight into the significance of genetics in human health and disease; however, many dental schools have not yet prioritized this area in their curricula. The understanding of genetic impact is blooming in dentistry. It will revolutionize patient treatment and handling in the future, enhancing diagnostic accuracy, patient care, and treatment outcomes. Given this, it is obvious that teaching dentistry students in basic and clinically applied genetics is a “need to” and “must be”, NOT a “nice to have” addition in universal dental education and curriculum regardless of nationality and ethnic background.

In our own newly established “Genetic Educators Network in Dentistry” among the Scandinavian sister countries, we have recognized a surprising difference in emphasis on the genetic courses in the dental curriculum between our very similar health and university systems. This insight led us to the current paper advocating for the inclusion of genetics education in all dental programs, aiming to prepare future dental professionals to meet the demands of the evolving “human genome era”. To facilitate this, we share suggestions for leaning goals and teaching material consisting of life-like cases aiming to inspire teachers and motivate dental students all over the world to embrace this highly relevant field.

## Figures and Tables

**Table 1:** **Goals and objectives for educational programs.** The learning goals are formulated according to the taxonomy of Bloom, applying the relevant verbs [27]. In the table, we have noted the relevant cases supporting each particular learning goal.

After the Course in Medical Genetics, the Students Should Be Able to:	Relevant Cases
1. Annotate and apply correct genetic terminology.	All
2. Explain how the developmental genes work at different stages of craniofacial development.	B, E
3. Name and describe inherited/genetic/congenital/multifactorial diseases of craniofacial development.	All
4. Explain the connection between genotype and phenotype in dental diseases, with emphasis on genetic heterogeneity.	All
5. Recognize inheritance patterns and evaluate if a tooth anomaly could be a part of a genetic syndrome.	A, B
6. Explain the chromosomal abnormalities and their effects on head and neck structures.	E
7. Explain the dynamics of inherited traits within the population, compare different populations, and calculate the frequency of disease or risk factor alleles.	B
8. Explain genetics in stratification of patients especially in multifactorial diseases.	D
9. Explain the genetic factors in somatic cancers and the heritability of the inherited cancers of the head and neck.	C
10. Explain the epigenetic factors and changes that take place in oral diseases as well as being conscious about the epigenetic effects of environmental factors.	D
11. Implement and analyze the molecular techniques of modern genetics and know which analyses to ask for in different circumstances.	All
12. Appraise the role of the dentist working as part of a health team for diagnosis, management, treatment, or prophylaxis of diseases with genetic involvement.	All
13. Recognize relevant genetic conditions and understand when and where to refer the patients.	All
14. Discuss and apply new developments and emerging topics in modern genetics as a preparation for the future.	All

## Data Availability

The original contributions presented in the study are included in the article/Supplementary Material, further inquiries can be directed to the corresponding author.

## Supplementary Materials

The following supporting information can be downloaded at: https://www.mdpi.com/article/10.3390/genes15121499/s1, Figure S1: A—Case on AI-Student handout; Figure S2: B—Case on HED–Student handout; Figure S3: C—Case on GS–Student handout; Figure S4: D—Case on MIH–Student handout; Figure S5: E—Case on dup9p–Student handout; Figure S6: Case B—1-HED Pedigree; Figure S7: Case B-2-EDA EDAR; Figure S8: Case D—MIH Pedigree; Figure S9: Case E-1—Array-CGH fetus_dup9p; Figure S10: Case E-2–Karyogram fetus der22t(9;22): Figure S11: Case E—3 Karyogram mother t(9;22).
