# Supplementary material for: Targeted Genetic Education in Dentistry in the Era of Genomics
Source: Genes (Basel). 2024 Nov 22;15(12):1499. doi: 10.3390/genes15121499 (PMC11675337; doi:10.3390/genes15121499)
Supplement: Supplementary file 1 [file genes-15-01499-s001.zip › Suppl fig 1 - A - Case on AI - student handout.pdf]

Case on Amelogenesis imperfecta (AI)

- Adam (age 10) is unhappy with his new adult teeth. They all have a rough surface, and he think they are somewhat yellow too.
- The dentist told him that it is an abnormal structure of the enamel that causes miscoloration
- Adam’s mother says that her brother and his son (her nephew) are both diagnosed with Amelogenesis imperfecta (AI)
- Adam is offered genetic screening of the genes *AMELX, ENAM, AMBN, MMP20* and *KLK4*
- Adam’s parents want to know the risk of AI for future children - can they do anything to prevent the condition and can it become even worse?
