# Supplementary material for: Targeted Genetic Education in Dentistry in the Era of Genomics
Source: Genes (Basel). 2024 Nov 22;15(12):1499. doi: 10.3390/genes15121499 (PMC11675337; doi:10.3390/genes15121499)
Supplement: Supplementary file 1 [file genes-15-01499-s001.zip › Suppl fig 2 - B - Case on HED - student handout.pdf]

Case on Hypohidrotic Ectodermal Dysplasia (HED)

- Pedigree of Helena (←) and her husband Eric. Helena is pregnant with their first baby (▪▪▪▪)
- The HED phenotype: agenesis, missing sweat glands, and thin hair, was found in Helena's brother and several of their relatives, and in Eric's sister
- Helena's sister shows weak symptoms of HED
- DNA sequencing results show missense DNA variants in *ED1* in Helena's brother and in *EDAR* in Eric's sister

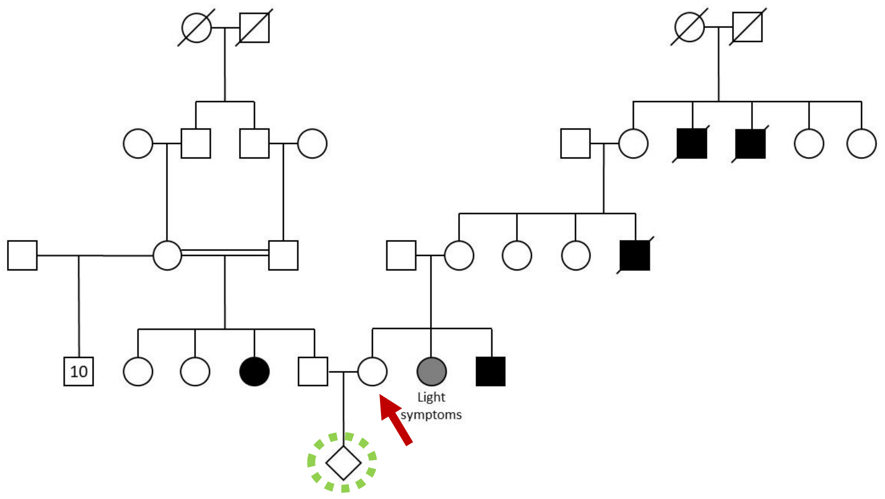

• Prevalence of HED in Eric's population	
• Autosomal recessive forms	• 1:100,000
• X-linked recessive forms	• 1:10,000
