# Supplementary material for: Targeted Genetic Education in Dentistry in the Era of Genomics
Source: Genes (Basel). 2024 Nov 22;15(12):1499. doi: 10.3390/genes15121499 (PMC11675337; doi:10.3390/genes15121499)
Supplement: Supplementary file 1 [file genes-15-01499-s001.zip › Suppl fig 3 - C - Case on GS - student handout.pdf]

Case on Nevoid Basal Cell Carcinoma, Gorlin syndrome (GS)

- Gina is a young woman suffering from a odontogenic keratocyst (OKC, jaw cyst) as well as several basal cell carcinomas (BCCs) of the skin indicating Gorlin syndrome
- Many of Gina’s family members had BCCs and her sister, father and paternal grandfather had BCCs and OKCs
- Gina’s big dream is to have children some day, but she is very worried that they might inherit her disease
- DNA sequence analysis of a blood sample from Gina revealed a heterozygous nonsense mutation in the *PTCH1* gene. Similar analysis of one of her BCCs revealed loss of heterozygosity in *PTCH1* as well as in a closely linked single nucleotide polymorphism (SNP)

	Blood	BCC
PTCH1	p.Q95* (c.283C>T)/normal	p.Q95*
SNP rs2277184	T/C	C
