# Supplementary material for: Targeted Genetic Education in Dentistry in the Era of Genomics
Source: Genes (Basel). 2024 Nov 22;15(12):1499. doi: 10.3390/genes15121499 (PMC11675337; doi:10.3390/genes15121499)
Supplement: Supplementary file 1 [file genes-15-01499-s001.zip › Suppl fig 4 - D - Case on MIH - student handout.pdf]

Case on Molar-Incisor Hypomineralization (MIH)

- Mike ( ) is 11 and has been suffering from Molar-Incisor Hypomineralization since he got his permanent teeth. It bothers him a lot, as some of his teeth have yellow-brownish spots and the enamel is fragile, leading to lots of caries. And he finds it very unfair that his identical twin Mark does not have this disease
- In Mike's family there are other cases of MIH as shown in the pedigree

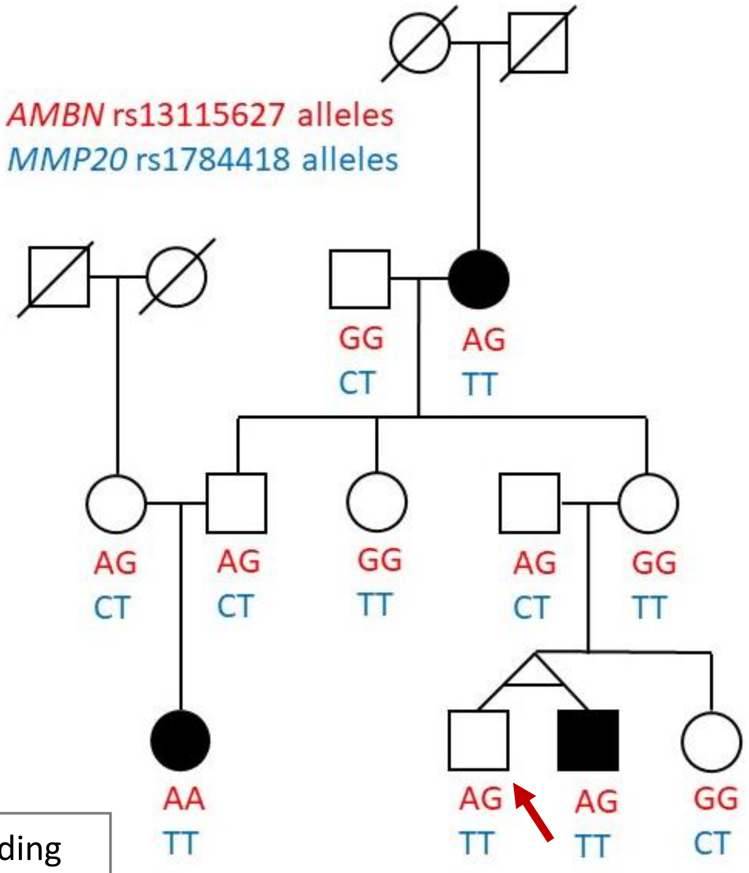

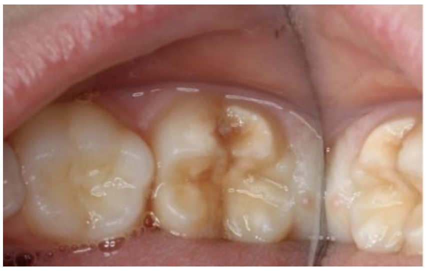

SNPs in the *AMBN* gene (encoding ameloblastin) and the *MMP20* gene (encoding enamelysin) were investigated. Overexpression of *AMBN* may cause MIH whereas homozygosity for loss-of-function variants in *AMBN* cause Amelogenesis Imperfecta
