# Supplementary material for: Targeted Genetic Education in Dentistry in the Era of Genomics
Source: Genes (Basel). 2024 Nov 22;15(12):1499. doi: 10.3390/genes15121499 (PMC11675337; doi:10.3390/genes15121499)

Case on chromosome 9p duplication and cleft lip and palate

- Ultrasound scan of a foetus shows cleft palate
- Follow-up array-CGH analysis of a chorion villus biopsy shows duplication of the short arm of chromosome 9
- Karyotyping of the fetus reveals an unbalanced translocation between chromosome 9 and 22

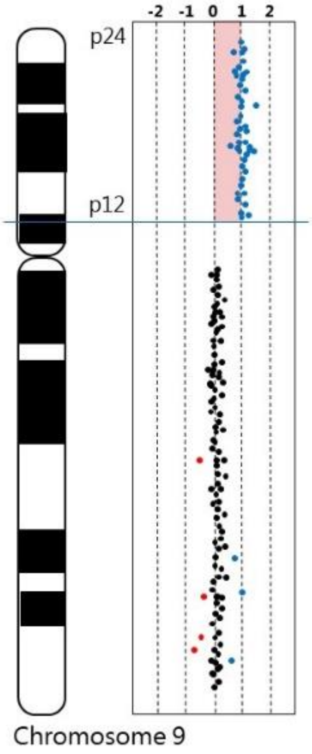

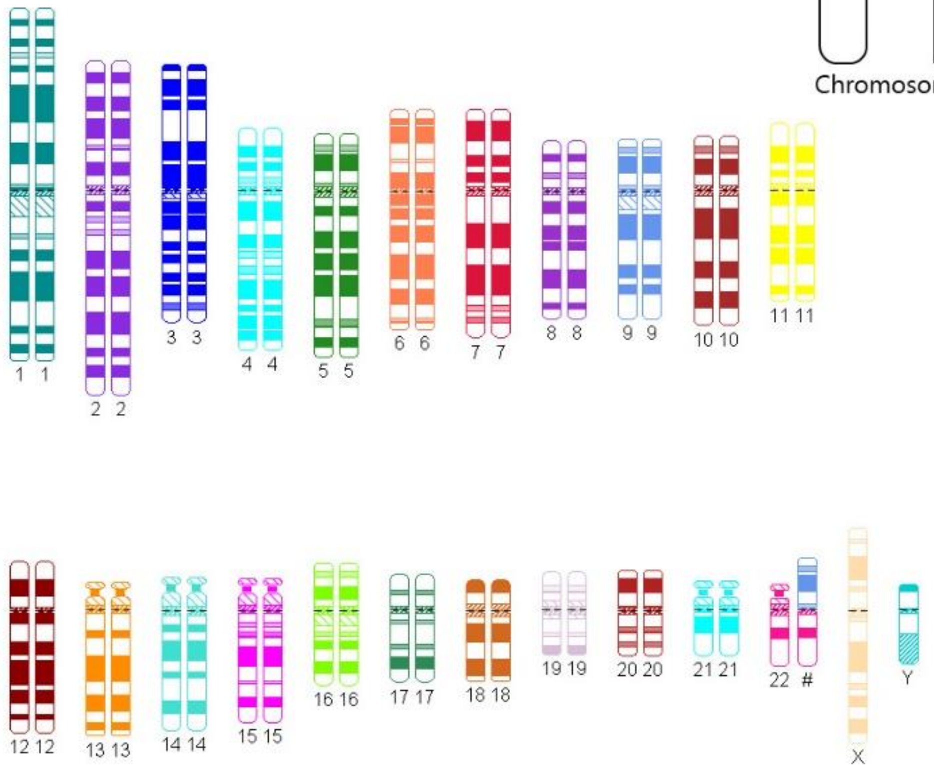

Supplement: Supplementary file 1 [file genes-15-01499-s001.zip › Suppl fig 5 - E - Case on dup9p - student handout.pdf]
