# Supplementary figures and images for: Targeted Genetic Education in Dentistry in the Era of Genomics
Source: Genes (Basel). 2024 Nov 22;15(12):1499. doi: 10.3390/genes15121499 (PMC11675337; doi:10.3390/genes15121499)

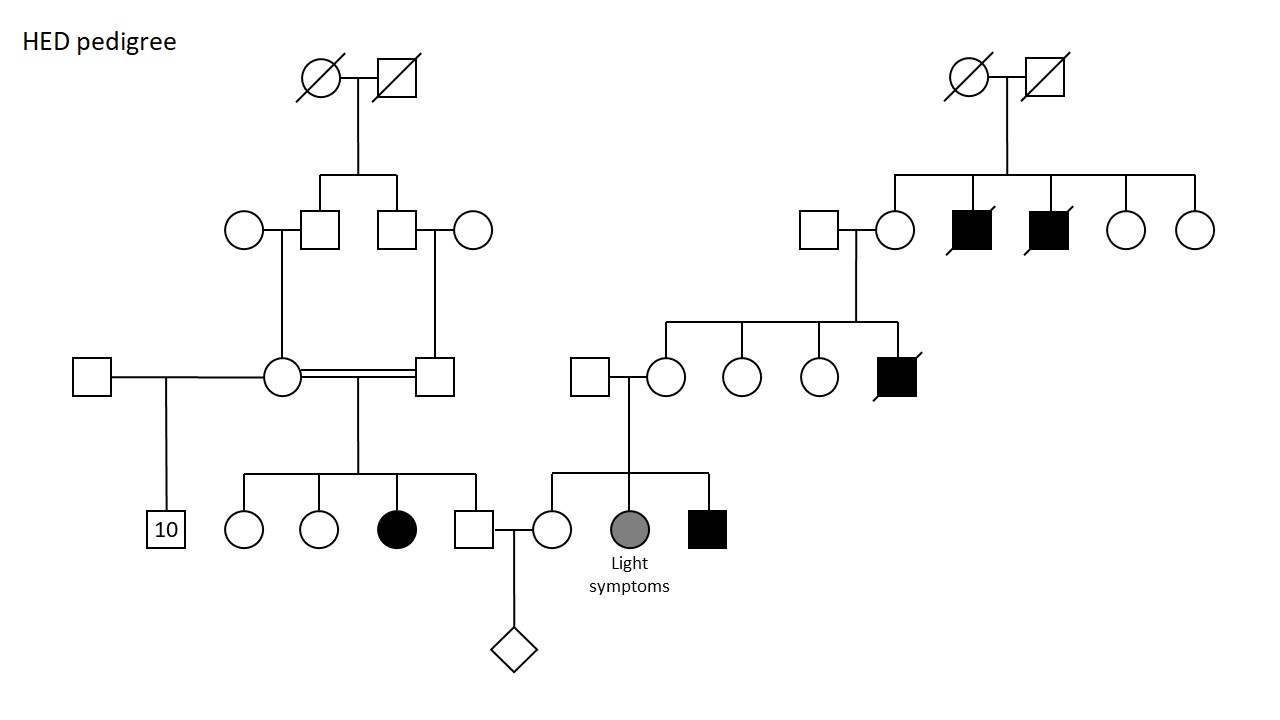

Supplement: Supplementary file 1 [file genes-15-01499-s001.zip › Suppl fig 6 - Case B-1 - HED pedigree.jpg]

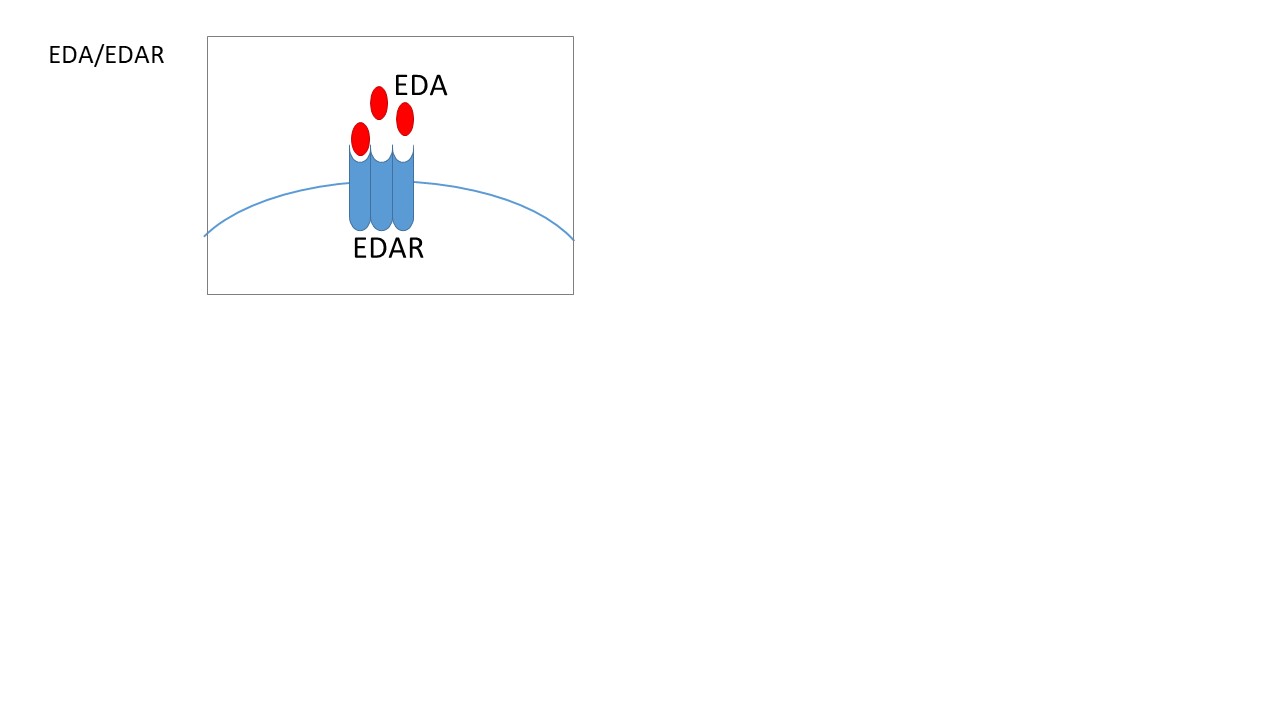

Supplement: Supplementary file 1 [file genes-15-01499-s001.zip › Suppl fig 7 - Case B-2 - EDA EDAR.jpg]

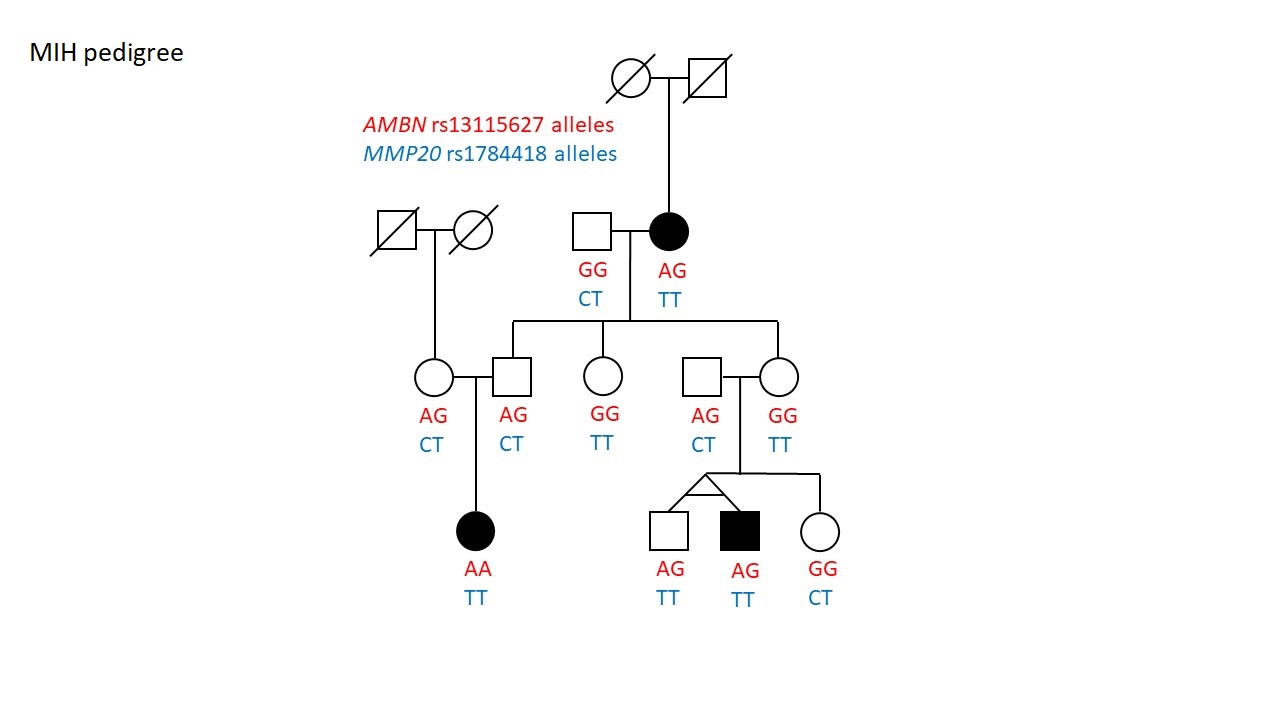

Supplement: Supplementary file 1 [file genes-15-01499-s001.zip › Suppl fig 8 - Case D - MIH pedigree.jpg]

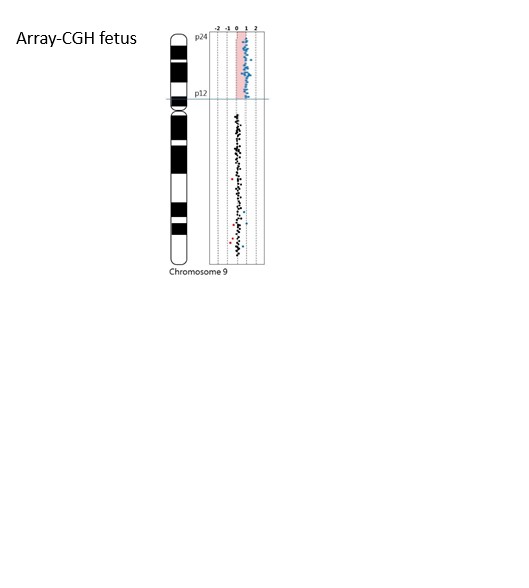

Supplement: Supplementary file 1 [file genes-15-01499-s001.zip › Suppl fig 9 - Case E-1 - array-CGH fetus dup9p.jpg]

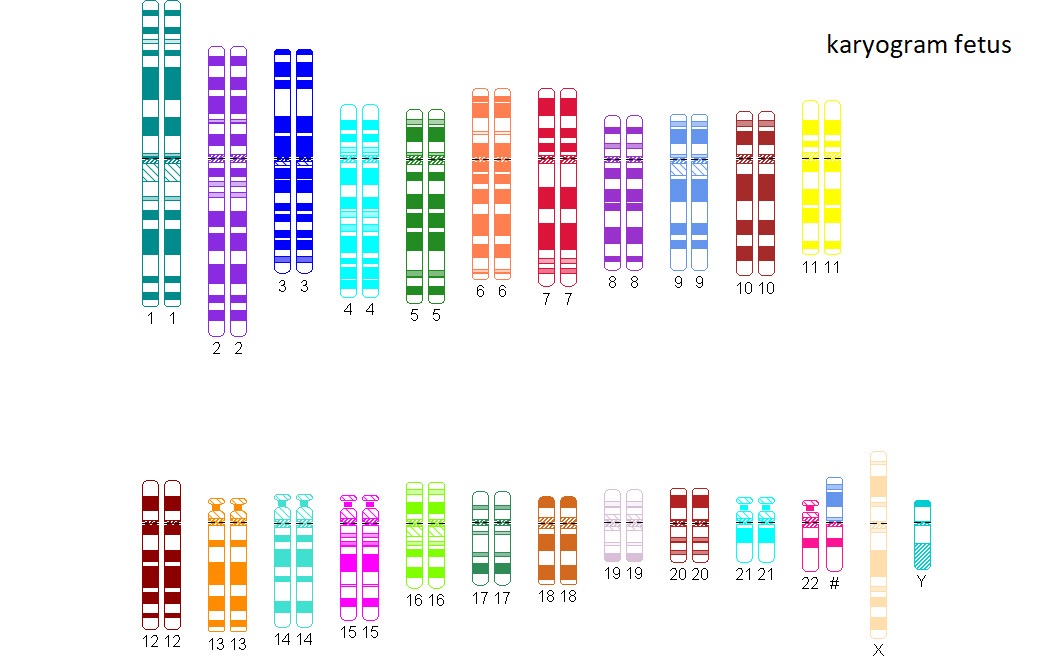

Supplement: Supplementary file 1 [file genes-15-01499-s001.zip › Suppl fig 10 - Case E-2 - karyogram fetus der(22)t(9,22).jpg]

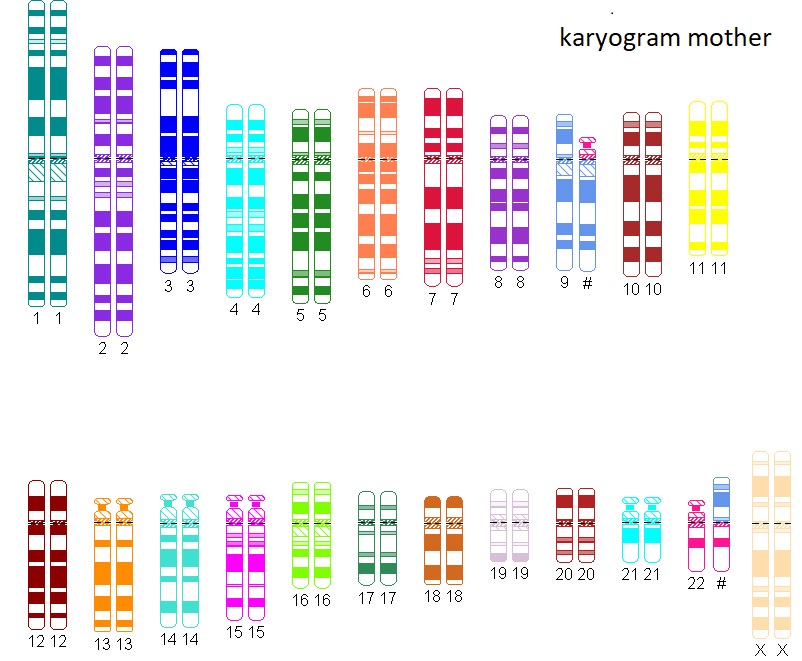

Supplement: Supplementary file 1 [file genes-15-01499-s001.zip › Suppl fig 11 - Case E-3 - karyogram mother t(9,22).jpg]
